# The symptom discounting effect: what to do when negative genetic test results become risk factors for alcohol use disorder

**DOI:** 10.1038/s41598-022-07452-5

**Published:** 2022-03-04

**Authors:** Woo-kyoung Ahn, Annalise M. Perricone

**Affiliations:** Department of Psychology, Yale University, 2 Hillhouse Ave., New Haven, CT 06511 USA

**Keywords:** Psychology, Health care

## Abstract

Most consumers of genetic testing for health conditions test negative, yet the psychological perils of this are hardly known. In three experiments (N = 2103) participants discounted repercussions of alcohol use disorder (AUD), after learning or imagining that they were not genetically predisposed to AUD. Such discounting can lead people to avoid treatment and to feel safe to continue or even increase their drinking, ironically turning the negative genetic feedback into a risk factor for AUD. Concerningly, the debriefing currently used by a direct-to-consumer genetic testing company failed to counteract this discounting among those already engaging in problematic drinking in all three studies. It was hypothesized that this discounting derives from not understanding the Causal Markov condition; once AUD symptoms are present, their ramifications remain the same regardless of whether genes or environmental factors caused the symptoms. Educating participants about this principle successfully mitigated the irrational discounting of threats of AUD.

## Introduction

Personalized genetic testing for a vulnerability to various health conditions has become widely available to the public, aimed at enhancing individually tailored prevention and treatment of illnesses. In 2018, over 75,000 genetic tests were available on the U.S. market with about ten new tests added daily^1^. The number of consumers of 23andMe, the most popular direct-to-consumer (DTC) genetic testing service offering health reports in the U.S., doubled from 2017 to 2018 and again from 2018 to 2019^2^. As genomic testing becomes increasingly prevalent (albeit to varying degrees across the world^3^), understanding its psychological impacts has become crucial.

### Irrationality of discounting threats of symptoms after testing negative

One unintended adverse consequence of genetic testing that was previously demonstrated is that laypeople perceive a genetically caused health condition as more permanent^4^, resistant to treatment^5^, and less controllable^6–9^, because they falsely believe that genes are immutable. Less known, however, are the perils of testing negative for genetic risks to health conditions.

Some researchers have warned that negative genetic feedback can induce false reassurance^10^. Indeed, upon learning that they lacked a genetic predisposition for obesity, laypeople in one study believed that they were less likely to develop obesity and discounted the importance of exercise and diet^11^. Yet, no previous studies have shown that the reassurance stemming from negative genetic test results is indeed *false* reassurance. In the aforementioned study, genetic predispositions for obesity were described as risk factors, so it was reasonable for participants to conjecture that if they tested negative for a genetic predisposition to obesity, they might not have had to worry about their exercise and diet as much as those who are genetically predisposed. Unlike previous studies, the current study presents the first empirical demonstration of the irrational beliefs caused by receiving negative genetic test results: people undermine the significance of symptoms of a condition that they already have simply because those symptoms are not genetically caused.

Reasoning in this way is irrational and non-normative according to Causal Bayesian Network models, which have dominated modeling of causal phenomena for the past three decades in Artificial Intelligence, Data Science, and Cognitive Science^12–15^. One of the most important assumptions underlying these models is the Causal Markov condition, which states that conditional on all its direct causes, an event is independent of all variables that are not direct causes or effects of that event. For instance, grass is slippery when it is wet, and grass can be wet because of rain or a sprinkler. Given this causal structure, once we know that grass is wet (i.e., conditional on the direct cause of being slippery), it is rational to infer that it would be slippery regardless of what caused the grass to become wet in the first place. That is, violating the Causal Markov condition (e.g., changing the likelihood that wet grass would be slippery depending on how it became wet) is considered irrational.

Similarly, genetic status should not change the ramifications of symptoms that are already present. For instance, if a person already consumes too many alcoholic beverages and cannot control their drinking, the ramifications of that specific symptom (e.g., its effect on one’s overall health and functioning at work or in interpersonal relationships) remains the same, regardless of whether the symptom was caused by genetic or non-genetic factors. Certainly, some environmentally caused symptoms may be more controllable than genetically caused ones, but that does not mean that the danger posed by the environmentally caused symptoms that one has already developed is less.

Yet, there are reasons to predict that the false reassurance elicited by the negative genetic test results can be irrational enough to violate the Causal Markov condition. First, previous studies using artificial stimuli have shown such violations^16,17^. Second, people judge genetically caused symptoms to be more serious^18^ and even falsely remember them as having been more severe^19^, meaning that symptoms not caused by genes are seen as more transient and trivial. Consequently, negative genetic test results would make people feel invulnerable even after symptoms develop, such that they might fail to seek out necessary healthcare services for the symptoms and/or downplay their negative downstream consequences. The current study offers the first empirical demonstration of such irrational beliefs.

### Counteracting symptom discounting

The implications of these findings for public health are significant as DTC genetic testing becomes increasingly available. Furthermore, a large majority of people who undergo genetic testing receive negative test results. Although consumers of DTC testing are encouraged to discuss their results with genetic counselors, many counselors are unwilling to do so based on the DTC results^20^, and thus developing effective debriefings that can immediately follow the provision of DTC results is crucial. To address this, the current study also tests two approaches to counteract irrational symptom discounting.

The first approach is modeled after materials currently used by 23andMe, the only DTC company in the U.S. with marketing authorization from the FDA^21^. When informing customers that they do not have certain genetic variants associated with a disease, the company provides a debriefing explaining that despite the customer’s negative results, they can still develop that disease because of environmental factors or untested genetic factors. Consumers could comprehend this kind of information, which was used as evidence during the process of obtaining Food and Drug Administration approvals for 23andMe’s genetic health risk test for various hereditary diseases (e.g., Alzheimer’s disease, breast cancer)^22,23^. Nonetheless, there are concerns that “some individuals may understand the limits of 23andMe testing as an intellectual matter and yet still derive false reassurance”^24^. Furthermore, as noted earlier, people tend to believe that non-genetically caused symptoms are less severe than genetically caused ones, so simply describing non-genetic causal factors may not be enough to prevent people from discounting the threats of symptoms. In particular, when a symptomatic person is highly motivated to trivialize those symptoms (e.g., when a person wants to rationalize their uncontrollable alcohol consumption), a more direct intervention explaining the irrational base of such discounting may be needed.

Thus, the current study also tested the second approach based on our theoretical insight that the discounting of symptoms is a violation of the Causal Markov condition. If so, in addition to reminding people about alternative causes, teaching participants about the Causal Markov condition could counteract the discounting. Thus, we developed and tested educational materials where the Causal Markov condition is explained in addition to the alternative-cause explanations. If this intervention removes the symptom discounting effect beyond what the alternative-cause explanations alone can do, it will also provide support for the idea that the symptom discounting effect partly stems from an irrational violation of the Causal Markov condition.

## Overview of methods

### Alcohol use disorder (AUD) as a test case

The target disorder used to test the symptom discounting effect and the two versions of the educational materials is AUD. The disorder is characterized by an inability to control one’s alcohol consumption, to the extent that one’s drinking causes clinically significant impairment or distress. AUD is among the most prevalent and undertreated psychiatric disorders in the developed world^25^. In the U.S. alone, excessive alcohol consumption is the third-leading cause of preventable death and is responsible for more than 95,000 deaths each year^26^. Yet a majority of individuals with AUD go without treatment. In 2019, only 7.2% of individuals with AUD received treatment^27^. The prevalence rate of AUD increased by 50% in the U.S. from 2001 to 2012^28^, and the COVID-19 pandemic has already caused a rise in rates of alcohol consumption, at least in the U.K.^29^ and Australia^30^.

Besides its serious public health threats, AUD is relevant for examining the effects of receiving genetic test results because of the rapid progress made towards understanding the genetic bases of AUD^31^. Indeed, although not FDA-approved, some DTCs have previously tested for a genetic vulnerability to alcohol dependence and alcohol flush response^32^.

AUD also provides an important test case for the violation of the Causal Markov condition, because AUD is an addiction. If one feels invulnerable to the consequences of AUD symptoms after learning that they are not genetically predisposed to AUD, one may start drinking even more alcohol. That is, the negative genetic test results can ironically turn into a risk factor for alcoholism.

Studying AUD as a target condition also provides a valuable opportunity to examine the symptom discounting effect among those who may be more susceptible to this effect, namely people who already show hazardous drinking patterns. A common impediment to treatment-seeking among people with substance use disorders is denial of the seriousness of one’s problem or of the need for treatment^33^. Therefore, people with problematic drinking patterns may be especially liable to discount the seriousness of AUD symptoms and more resistant to counteracting measures.

Thus, at the beginning of each of the current studies, participants completed the Alcohol Use Disorders Identification Test (AUDIT)^34^, a self-report measure of AUD symptomatology developed by the World Health Organization (WHO). Using the WHO guidelines, those scoring at least 8 out of a possible 40 points were categorized as “problem-drinkers.” Then, the symptom discounting effect as well as the effects of the educational materials were examined separately for problem-drinkers and for non-problem-drinkers, in order to test whether problem-drinkers in particular show resistance to the intervention. Doing so also provides clinical utility because the AUDIT cutoff score of 8 has been found to provide the greatest clinical sensitivity and specificity for detecting hazardous drinking behavior^35,36^. Furthermore, this cutoff is frequently used by clinicians when identifying those engaging in problem-drinking, and thus, separately examining the symptom discounting effect and the efficacy of educational materials among those who engage in harmful drinking can provide information that will be more useful in clinical settings.

### Experimental manipulations of perceived genetic status

Three studies experimentally manipulated people’s thoughts about their genetic status for AUD. This was done by measuring participants’ perceptions of the ramifications of AUD symptoms before and after they were experimentally led to believe (Study 1) or imagined (Studies 2 and 3) that they lacked a genetic risk for AUD.

We used the experimental manipulations rather than examining the impact of receiving real-life genetic testing results for several reasons. First, as of 2022, there are no FDA-approved DTC testing for AUD genetic risks. Even if they soon become available, correlational studies based on real-life genetic testing results cannot discern whether the symptom discounting effect, if obtained, is due to the genetic feedback per se or to actual genetic differences which may correlate with other factors leading to the discounting effect (e.g., family environments, self-control). If the symptom discounting effect arises from these confounds rather than the genetic feedback per se, there would be no need to develop counteracting measures in relation to the genetic feedback. Experimentally manipulating feedback while randomizing the actual genetic differences allows us to avoid these confounds.

Specifically, Study 1 used a sham biochemical test of participants’ saliva, described to them as a test of their genetic susceptibility to AUD. (Sect. 1 of the Supplementary Information details the extensive measures taken to address ethical concerns with using this method.) In Studies 2 and 3, participants were asked to imagine that they received test results indicating that they lacked a genetic predisposition to AUD. Although Study 1 uses a more ecologically valid method, as in previous studies using this same method^7,11^, many participants had to be eliminated because they were understandably dubious about the validity of the “genetic feedback” provided in a psychology experiment. Yet, this elimination could inflate the likelihood of detecting a violation of the Causal Markov condition since those more skeptical participants might also have been less likely to violate that condition. By using the “pretend genetic feedback” manipulation and avoiding the potential selection bias, Studies 2 and 3 complement Study 1.

To measure their perception of the seriousness or downstream effects of AUD symptoms, participants in all three studies were prompted to imagine that they had AUD symptoms (i.e., spending a lot of time drinking alcoholic beverages and drinking more than intended). They then rated the ramifications of AUD symptoms (e.g., urgency to seek professional help, need to make changes in drinking behavior, extent to which the symptoms harm one’s functioning) before and after they were led to believe in or imagined the absence of the genetic predisposition. It was hypothesized that after receiving or imagining the negative test results, participants’ perception of the danger of AUD symptoms would be reduced compared to baseline.

Across the three experiments, the effects of educational materials were tested. All three studies tested the effect of the materials explaining the existence of alternative causes for AUD. Study 3 tested the effect of providing educational materials explaining the Causal Markov condition.

The Institutional Review Board of Yale University has approved all three studies. All experiments were performed in accordance with relevant guidelines and regulations, and all the participants provided their informed consent.

## Study 1

To mimic the direct-to-consumer genetic feedback, Study 1 used a sham saliva test, a method used in previous research^7,11,37^, leading people to believe that they lacked a genetic predisposition to AUD. All methods were approved by the Institutional Review Board, and details of the protection of participants are explained in the Supplementary Information. Study 1 also tested the type of educational materials used by 23andMe, reminding people about causes of AUD, other than the tested genes.

### Methods

#### Participants and recruitment

Participants were U.S. adults recruited by the survey research firm YouGov. The data collection took place across three phases because of the lower-than-expected yield of problem-drinkers in the first phase. In the first phase, a preliminary survey was used to exclude ineligible participants who do not drink and do not ever intend to drink alcoholic beverages. A total of 267 non-problem-drinkers and 50 problem-drinkers completed the survey. In the second and third phases, targeted to recruit problem-drinkers, participants first completed the AUDIT, and those scoring at least 8 were recruited. In the second phase, 44 participants who were validated again to be problem-drinkers during the main study completed the experiment, and the process was halted due to the challenge of producing and delivering the saliva testing kits through YouGov during the pandemic. Seven months after the second phase, using the same recruitment methods, the third phase of recruitment took place, and an additional 161 participants completed the experiment.

Of 522 participants who completed the survey, 11.3% incorrectly completed the saliva test procedure, 0.03% misunderstood the genetic feedback, and 33.5% did not agree that the genetic feedback they received was valid, failing the manipulation check (see below). There was no significant difference in AUDIT scores between those who did not agree that the genetic feedback was valid (*M* = 7.16, *SD* = 6.00) and those who did (*M* = 8.12, *SD* = 7.43), *t*(452) = 1.39, *p* = 0.165. Using the same exclusion criteria as in previous studies that employed a similar saliva-test methodology^7,11^, those who misunderstood the genetic feedback or did not agree that it was valid were excluded from the analyses reported below, yielding a total of 301 participants. Among these, 159 were non-problem-drinkers (58.5% female, mean age = 50.5 [SD = 16.9], 75.5% white, 34.6% with a bachelor’s or higher degree) and 142 were problem-drinkers (45.8% female, mean age = 50.2 [SD = 13.9], 81.7% white, 41.6% with a bachelor’s or higher degree).

#### Design

**Figure 1:**
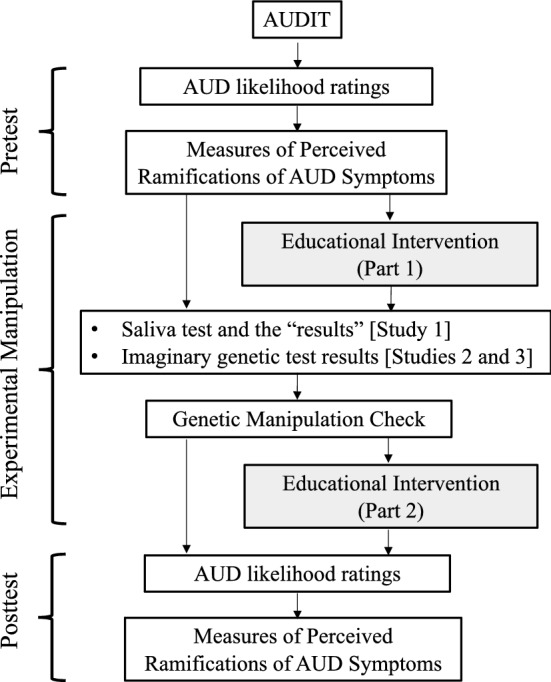
Summary of design and procedures of Studies 1–3. Boxes shaded in grey represent procedures that were only included in the Alternative-Cause condition (Studies 1–3) and the Causal-Markov condition (Study 3). AUDIT = Alcohol Use Disorders Identification Test.

Study 1 was a 2 X 2 factorial design as summarized in Fig. 1. The first factor was time-point; all participants indicated how they felt about AUD symptoms before and after receiving the genetic “test results”. As a second factor, participants were randomly assigned to either the No-Education (80 non-problem-drinkers and 68 problem-drinkers) or the Alternative-Cause (79 non-problem-drinkers and 74 problem-drinkers) condition.

#### Measures of perceived ramifications of AUD symptoms

To measure how participants perceived the seriousness, urgency, and other ramifications of AUD symptoms, we developed 10 items. Each of these items described a hypothetical scenario where the participant or a family member was exhibiting AUD symptoms, as defined in the Diagnostic and Statistical Manual of Mental Disorders^38^, and then measured how serious and impactful the participant would consider the symptoms to be. These items, shown in Table 1, were organized in four blocks.

**Table 1:** Measures of perceived ramifications of AUD symptoms used in Studies 1–3.

Block Name and *Prompt*	Items and Response Options
“Self” Block: *Suppose that you begin to spend a lot of time drinking alcoholic beverages, and you notice that you sometimes end up drinking more than you intended*	1. How serious of a problem would this be? (From 0 “not at all serious” to 10 “as serious as the most serious problem you can imagine”) 2. How confident are you that you could stop these behaviors on your own, without professional help or treatment? (From 0 “not at all confident” to 10 “extremely confident”; reverse-coded) 3. How urgent would it be for you to seek professional treatment for this? (From 0 “not at all urgent” to 10 “extremely urgent”)
“Family Member” Block: *Suppose that a close family member of yours, who is biologically related to you, begins to spend a lot of time drinking alcoholic beverages, and this family member sometimes ends up drinking more than he or she intended*	4. How serious of a problem would this be? (From 0 “not at all serious” to 10 “as serious as the most serious problem you can imagine”) 5. How confident are you that this family member could stop these behaviors on his or her own, without professional help or treatment? (From 0 “not at all confident” to 10 “extremely confident”; reverse-coded) 6. How urgent would it be for this family member to seek professional treatment for this? (From 0 “not at all urgent” to 10 “extremely urgent”)
“Upstream” Block: *Suppose that you begin to spend a lot of time drinking alcoholic beverages, and you notice that you sometimes end up drinking more than you intended*	Please rate your agreement with the following statement on a scale from 0 (totally disagree) to 10 (totally agree): 7. I would really need to make changes in my drinking. (From 0 “totally disagree” to 10 “totally agree”) 8. I would believe that I should be doing things to cut down or stop drinking. (From 0 “totally disagree” to 10 “totally agree”)
“Downstream” Block: *Suppose that you begin to spend a lot of time drinking alcoholic beverages and you notice that you sometimes end up drinking more than you intended*	Please rate your agreement with the following statement on a scale from 0 (totally disagree) to 10 (totally agree): 9. My drinking would cause a lot of harm. (From 0 “totally disagree” to 10 “totally agree”) 10. My drinking would interfere with my work AND/OR social functioning. (From 0 “totally disagree” to 10 “totally agree”)

The order of the “self” and “family member” block pair and the “upstream” and “downstream” block pair was counterbalanced, and the order within each pair was counterbalanced. The item numbers were not presented to the participants. Supplementary Information shows the differences between the pre-test and post-test ratings for each item across the No-Education conditions of Studies 1–3.

The “self” and “family member” blocks each contained three items measuring how participants would react if they themselves or a biologically related family member began exhibiting AUD symptoms. The items concerning a family member were created for two reasons. First, family members often play a pivotal role for one another when dealing with symptoms of AUD^39,40^, so there are immediate clinical implications if a person tests negative in AUD genetic testing and discounts the seriousness of their biologically related family members’ AUD symptoms. Second, negative genetic test results may cause one to feel invulnerable about their own AUD symptoms because of the ubiquitous overconfidence bias^41^, but they may be more cautious when reasoning about their family members. Thus, these items provide a stronger test of the symptom discounting effect.

The “upstream” and “downstream” blocks contained, respectively, two items measuring what kind of reactions the symptoms warranted and two assessing the perceived downstream consequences of alcohol consumption. The order of the “self” and “family member” block pair and the “upstream” and “downstream” block pair was counterbalanced, and the order within each pair was counterbalanced.

#### Alternative-cause educational materials

The educational materials for the participants in the Alternative-Cause condition were constructed based on information presented to people receiving negative genetic test results from the DTC genetic testing company 23andMe. The educational materials explained in a series of slides that AUD risk results from both genetic and non-genetic factors and that a person could have other genetic risk factors for AUD not assessed by the test participants took. These educational materials were presented in two parts, and the “genetic test results” were provided between the two parts, replicating the 23andMe debriefing procedure where the first part is presented as a required tutorial before receiving the health reports and the second part is presented after the health reports.

Part 1 of our educational materials began with a brief introduction to alcoholism: “Alcoholism, also known as alcohol use disorder, often runs in families. Scientific studies have shown that genes influence a person's risk of developing alcoholism. In the following screens, you will read some explanations about the genetics of alcoholism.” Then, the materials provided an explanation of what genetic variants and genetic risk factors are and stated that certain genetic variants can increase a person’s risk of developing alcoholism. Next, the materials defined alcoholism as “a disorder that results when a person engages in uncontrolled, chronic drinking and becomes physically and emotionally dependent on alcohol.” Subsequently, the materials explained that although genetic risk factors can increase a person's likelihood of developing alcoholism, even people without genetic risk factors for alcoholism can be at risk of developing the disorder because of non-genetic factors, such as behavior and environment. It was also emphasized that not everyone with a genetic risk factor for alcoholism will develop the disorder. The educational materials also described how some genetic tests might be able to determine whether one has an increased risk of developing alcoholism, but how one may be able to control this risk by managing other risk factors with the help of their doctor, even if one tests positive for a genetic risk of developing alcoholism.

Part 2 of the educational materials was presented to the participants in the Alternative-Cause condition after they were told that they lacked a genetic predisposition to alcoholism (see below for details). They were told that although their test results showed that they were NOT genetically predisposed to alcoholism, the test did not look for every genetic risk factor that increases a person’s likelihood of developing alcoholism. They were then reminded that there are other genetic and non-genetic risk factors for alcoholism, and were provided with specific examples (e.g., other genetic variants, lifestyle, age). It was stated “as such, you may still be at risk of developing alcoholism because of other genetic or non-genetic factors.” The educational materials ended with, “Alcoholism risk is widespread and affects people with many different genetic makeups”.

The verbatim version of these alternative-cause educational materials can be available by contacting the corresponding author.

#### Procedures

Participants were mailed a parcel containing the “testing kit” that was needed to complete the sham saliva test, with instructions stating that they should not open the kit until instructed and detailing how to access the study via Qualtrics. After reading the informed consent, which noted that they had the option to withdraw from the study at any time, participants received instructions about how to use an “Exit Study” link so that they could be properly debriefed and compensated if they withdrew (See Supplementary Information, Sect. 1 for details).

Study procedures, summarized in Fig. 1, were administered using Qualtrics.com online data-collection software. Participants first completed the AUDIT, and those scoring at least 8 out of a possible 40 points were categorized as “problem-drinkers.” The No-Education (*M* = 7.77, *SD* = 7.10) and the Alternative-Cause (*M* = 8.46, *SD* = 7.73) conditions did not significantly differ in AUDIT scores, *t*(299) = 0.80, *p* = 0.423, *d* = 0.09.

All participants completed the pre-test measures. The first item measured participants’ beliefs about their likelihood of developing alcoholism. Specifically, participants were asked to rate the likelihood from 0 to 100% that they would become an alcoholic if they had 3–4 alcoholic drinks almost every day. Even if likelihood ratings are lower at post-test (i.e., after receiving the negative “genetic feedback”), it is arguable whether such a decrease is necessarily irrational. As such, this measure was not the main focus of the study, but instead was included to provide converging evidence. Then, participants were presented with 10 items measuring their perception of the ramifications of AUD symptoms, which were the main dependent measure of the study.

After the pre-test measures, participants in the No-Education condition carried out the “saliva test.” To make the cover story for the test credible, participants were told that the test would assess their salivary levels of the enzyme aldehyde dehydrogenase, which was described as revealing whether one is genetically predisposed to AUD. Then participants were instructed to open the “testing kit”, rinse their mouths with the mouthwash contained in the kit (which, unbeknownst to participants, had been mixed with glucose) for seven seconds, insert the test strip (which was actually a glucose test strip) under their tongues for 10 s, and then wait 45 s. The glucose in the mouthwash caused a blue square on the test strip to change to a brownish green color. Participants were asked to select whether the color of the square changed to “Brown or Green,” “Red or Pink,” or “White.” Those who did not select “Brown or Green” were classified as incorrectly following the procedure and excluded from analyses. Participants were then told that the selected color indicated that they were not genetically predisposed to alcoholism. A manipulation check item then asked whether the results showed that they were genetically predisposed to AUD, and only those who correctly responded were included in analyses.

Participants in the Alternative-Cause condition also carried out the same saliva test, but they received Part 1 of the alternative-cause educational materials first. After completing the saliva test, receiving the negative “test results”, and the experimental manipulation check as in the No-Education condition, they received Part 2 of the alternative-cause educational materials.

Afterwards, participants in both conditions completed the post-test alcoholism likelihood assessment and measures of the perceived ramifications of AUD symptoms, which were the same as the pre-test measures. After providing basic demographic information but before receiving the debriefing, participants rated their agreement with a statement that the test "gave accurate and reliable information about my genetic makeup" on a 5-point scale (1: “Strongly Disagree”—5: “Strongly Agree”). Participants who did not provide a rating of 4 or 5 (“Agree” or “Strongly Agree”) were excluded from analyses as was done in the previous studies using similar saliva test methods^7,11^. Among the participants whose data was reported in the analyses, the No-Education (*M* = 4.35, *SD* = 0.479) and the Alternative-Cause (*M* = 4.33, *SD* = 0.471) conditions did not significantly differ in the credibility ratings, *t*(299) = − 0.449, *p* = 0.654, *d* = 0.04.

Finally, participants completed an extensive and detailed debriefing process, carefully developed in consultation with the Yale University IRB. (See Supplementary Information, Sect. 1.)

## Results

Cronbach’s alpha was high for the 10 pre-test items (0.86) and the 10 post-test items (0.90) measuring the perceived ramifications of AUD symptoms. Thus, each set was averaged for each participant to compute pre-test and post-test AUD symptom ramification scores such that lower scores indicate more discounting of AUD symptom severity. In all the subsequent analyses, a mixed-design ANOVA involving one within-subjects factor (timepoint) and one between-subjects factor (condition) was used to test whether the AUD symptom discounting effect (i.e., decrease from pre-test to post-test ratings) was moderated by the educational materials.

### Non-problem-drinkers

**Figure 2:**
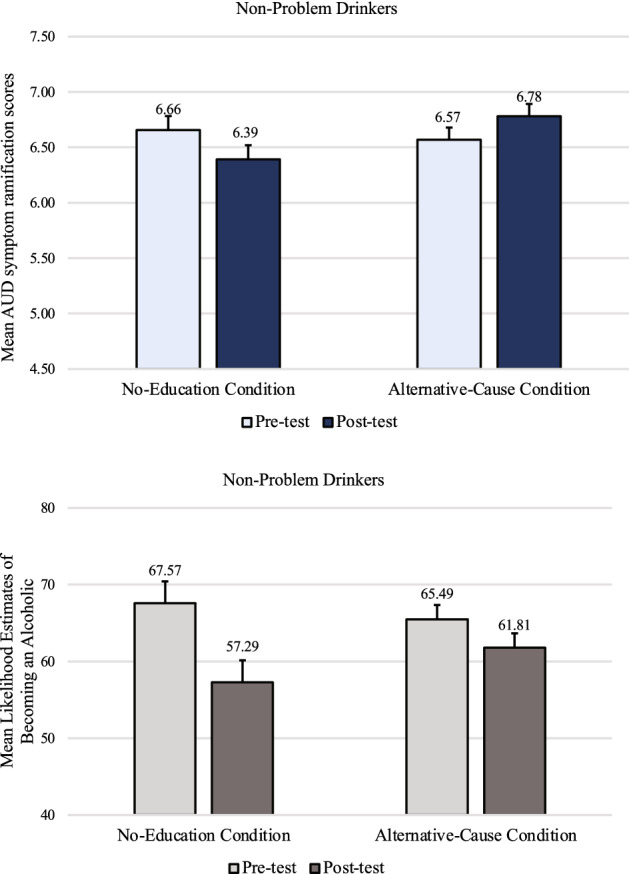
Non-problem-drinkers’ mean AUD symptom ramification scores and mean likelihood estimates of becoming an alcoholic in Study 1, by condition at pre-test and post-test. Error bars represent *SEM* ^*pairedDiff*^*.*

A mixed-design timepoint X condition ANOVA examining AUD symptom ramification scores among non-problem-drinkers (top panel of Fig. 2) revealed that the only significant effect was the interaction, *F(*1, 157) = 7.86, *p* = 0.006, η_p_^2^ = 0.05.

Paired *t*-tests showed that non-problem-drinkers who did not receive any educational materials discounted the seriousness and urgency of AUD symptoms after the genetic feedback (*M* = 6.39, *SD* = 1.94) compared to before (*M* = 6.66, *SD* = 1.83), *t*(79) = − 2.06, *p* = 0.043, *d* = 0.23. When non-problem-drinkers received the educational materials about non-genetic causes, there was no evidence of this discounting effect. In the Alternative-Cause condition, participants actually treated AUD symptoms more seriously and urgently at post-test (*M* = 6.78, *SD* = 1.84) than at pre-test (*M* = 6.57, *SD* = 1.81), *t*(78) = 1.91, *p* = 0.060, *d* = 0.21.

Participants’ ratings of their likelihood of becoming an alcoholic if they began having 3–4 alcoholic beverages a day were also examined (bottom panel of Fig. 2). A mixed-design timepoint X condition ANOVA on the AUD likelihood ratings revealed that non-problem-drinkers felt significantly less likely to become an alcoholic after they were told that they lack a genetic predisposition (*M* = 59.55, *SD* = 32.56) than before (*M* = 66.53, *SD* = 32.26), *F(*1, 156) = 16.73, *p* < 0.001, *f* = 0.32. There was no main effect of condition, *p* = 0.802, η_p_^2^ < 0.01, but there was a marginally significant interaction effect, *F(*1, 156) = 3.733, *p* = 0.055, η_p_^2^ = 0.023, because while participants in both conditions rated their likelihood of becoming an alcoholic as lower at post-test than at pre-test, the decrease was greater in the No-Education condition (mean post-test vs. pre-test difference of 10.29, *t*(78) = 3.59, *p* < 0.001, *d* = 0.41) than in the Alternative-Cause condition (mean post-test vs. pre-test difference of 3.68, *t*(78) = 1.98, *p* = 0.051, *d* = 0.22).

### Problem-drinkers

**Figure 3:**
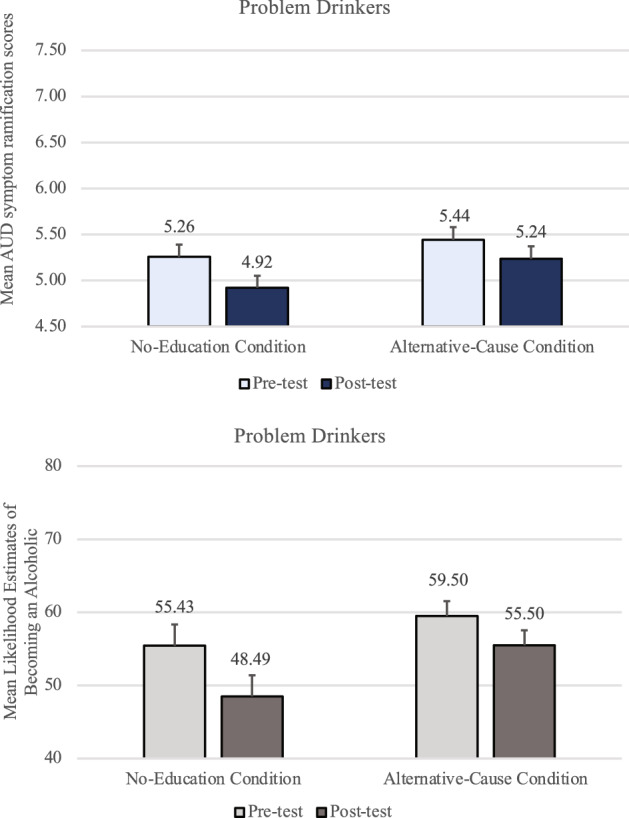
Problem-drinkers’ mean AUD symptom ramification scores and mean likelihood estimates of becoming an alcoholic in Study 1, by condition at pre-test and post-test. Error bars represent *SEM* ^*pairedDiff*^*.*

A mixed-design timepoint X condition ANOVA on AUD symptom ramification scores revealed no main effect of condition, *F*(1, 140) = 0.63, *p* = 0.430, η_p_^2^ < 0.01. Unlike with non-problem-drinkers, there was no significant interaction effect, *F(*1, 140) = 0.47, *p* = 0.494, η_p_^2^ < 0.01. Instead, there was only a significant main effect of timepoint; AUD symptom ramification scores were significantly lower after the genetic feedback (*M* = 5.09, *SD* = 2.00) than before (*M* = 5.36, *SD* = 2.00), *F*(1, 140) = 8.30, *p* = 0.005, *f* = *0.23,* regardless of whether problem-drinkers received the educational materials on the existence of alternative causes (top panel of Fig. 3).

Problem-drinkers’ ratings of their likelihood of becoming an alcoholic (bottom panel of Fig. 3) also mirrored their AUD symptom ramification scores. A mixed-design timepoint X condition ANOVA revealed that problem-drinkers felt significantly less likely to become an alcoholic after being told that they lack a genetic predisposition (*M* = 52.14, *SD* = 32.84) than before (*M* = 57.55, *SD* = 32.07), *F*(1, 140) = 9.86, *p* = 0.002, *f* = 0.25. There was no main effect of condition, *p* = 0.285, η_p_^2^ < 0.01, nor an interaction effect, *p* = 0.400, η_p_^2^ < 0.01.

### Other analyses

See Supplementary Information, Sect. 2 for analyses comparing non-problem-drinkers and problem-drinkers, which explore whether there are differences between these two groups in recognizing the symptom ramifications as well as in the efficacy of the educational materials.

## Study 2

Study 2 attempts to replicate Study 1 using a different method. In Study 1, a substantial number of participants who did not endorse the validity of the saliva test had to be excluded, which could have resulted in a selection bias, if those participants who were dubious of the test were also less likely to violate the Causal Markov condition. Instead of a sham saliva test, participants in Study 2 were asked to imagine that they lacked genetic risks for alcoholism. Study 2 was preregistered via the Open Science Framework repository (see osf.io/y36pe).

### Methods

#### Participants

Participants were a sample of U.S. adults recruited through Amazon.com’s Mechanical Turk (MTurk) platform in exchange for compensation. Of 519 participants who initially completed the study, 51 were excluded from analyses because they failed an experimental manipulation check asking them to confirm the type of results they were asked to imagine having received (i.e., positive or negative), and 7 were excluded for failing to complete all dependent measures. The final sample consisted of the remaining 461 participants. Among these, 319 were non-problem-drinkers (51.6% female, mean age of 36.1 [*SD* = 11.8], 78.2% white, 54.7% with a bachelor’s or higher degree) and 142 were problem-drinkers (40.7% female; mean age of 35.3 [*SD* = 10.7], 80.0% white, 45.0% with a bachelor’s or higher degree).

#### Design and procedures

The design of Study 2 was identical to Study 1. Participants were randomly assigned to either the Alternative-Cause condition (*n* = 232) or a No-Education condition (*n* = 229). The two conditions did not differ significantly in AUDIT scores (*M* = 6.76, *SD* = 6.30 for the No-Education condition; *M* = 6.34, *SD* = 5.50 for the Alternative-Cause condition), *t*(459) = 0.76, *p* = 0.447, *d* = 0.07.

Study procedures were identical to Study 1 except for the following. Instead of the saliva test procedure and the feedback from the saliva test used in Study 1, participants were instructed to imagine that they lacked a genetic predisposition to AUD. All participants were told, “Suppose you received, as a free gift, the chance to undergo genetic testing performed by a highly reputable company. You sent in the testing kit following the instructions. Several days later, you receive the results. In the report, it is stated that you are NOT genetically predisposed to alcoholism.” Immediately following this prompt, participants were asked: “According to the test, which of the following statements is correct?” The response options were “You ARE genetically predisposed to alcoholism” and “You are NOT genetically predisposed to alcoholism”; only participants who correctly selected the latter option were included in analyses. Participants then completed the post-test measures as in Study 1 except that on each page, they were reminded to assume that they lacked a genetic risk ƒor AUD. This reminder was added because the experimental manipulation of merely imagining the genetic status might not be as memorable as receiving the saliva test results in Study 1.

## Results

Cronbach’s alpha was high for both the 10 pre-test items (0.88) and the 10 post-test items (0.92). Thus, AUD symptom ramification scores for each participant were computed as in Study 1.

### Non-problem-drinkers

**Figure 4:**
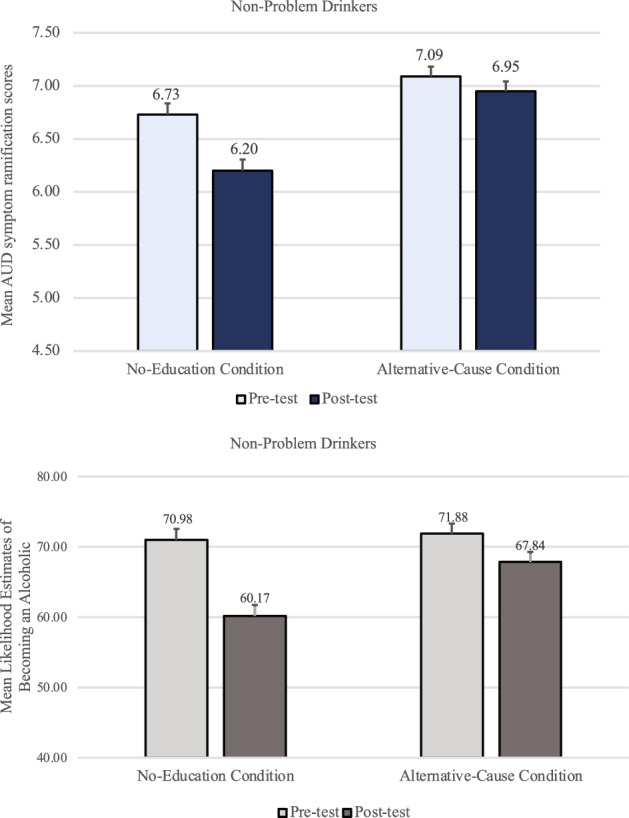
Non-problem-drinkers’ mean AUD symptom ramification scores and mean likelihood estimates of becoming an alcoholic in Study 2, by condition at pre-test and post-test. Error bars represent *SEM*^*pairedDiff*^*.*

A mixed-design timepoint X condition ANOVA examining AUD symptom ramification scores among non-problem-drinkers revealed that there was a significant main effect of timepoint, *F(*1, 317) = 27.59, *p* < *0.0*01, *f* = 0.29, and a significant main effect of the educational materials, *F(*1, 317) = 9.30, *p* = 0.002, η_p_^2^ = 0.03, but these main effects were qualified by a significant interaction effect, *F(*1, 317) = 10.14, *p* = 0.002, η_p_^2^ = 0.03 (top panel of Fig. 4). A paired *t*-test in the No-Education condition showed that non-problem-drinkers discounted the seriousness and urgency of AUD symptoms after the genetic feedback (*M* = 6.20, *SD* = 1.83) compared to before (*M* = 6.73, *SD* = 1.66), *t*(156) =  − 5.63, *p* < *0.0*01, *d* = 0.45. In the Alternative-Cause condition, however, there was no significant difference between pre-test (*M* = 7.09, *SD* = 1.68) and post-test scores (*M* = 6.95, *SD* = 1.72), *t*(161) =  − 1.56, *p* = 0.122, *d* = 0.12. Thus, among non-problem-drinkers, this analysis revealed no evidence of a symptom discounting effect in the Alternative-Cause condition, as in Study 1.

Participant ratings of their likelihood of becoming an alcoholic if they had 3–4 alcoholic beverages a day were also examined (bottom panel of Fig. 4). A mixed-design timepoint X condition ANOVA revealed that non-problem-drinkers felt significantly less likely to become an alcoholic after they were told that they lacked a genetic predisposition (*M* = 64.06, *SD* = 29.04) than before (*M* = 71.43, *SD* = 29.24), *F*(1, 316) = 48.93, *p* < 0.001, *f* = 0.39. There was no main effect of condition, *p* = 0.166, η_p_^2^ < 0.01, but there was a significant interaction effect, *F*(1, 316) = 10.21, *p* = 0.002, η_p_^2^ = 0.031, because while both conditions’ likelihood ratings went down after the feedback, the decrease was greater in the No-Education condition (mean post-test vs. pre-test difference of 10.81) than in the Alternative-Cause condition (mean post-test vs. pre-test difference of 4.04, *t*(316) = 3.20, *p* = 0.002, *d* = 0.36).

### Problem-drinkers

**Figure 5:**
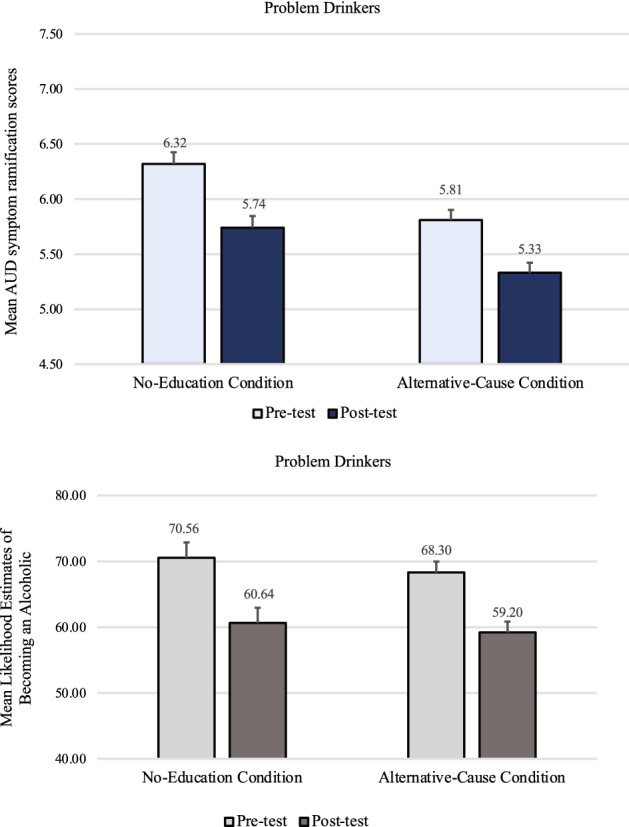
Problem-drinkers’ mean AUD symptom ramification scores and mean likelihood estimates of becoming an alcoholic in Study 2, by condition at pre-test and post-test. Error bars represent *SEM*^*pairedDiff*^.

A mixed-design timepoint X condition ANOVA examining AUD symptom ramification scores among problem-drinkers revealed that AUD symptom ramification scores were significantly lower after the genetic feedback (*M* = 5.54, *SD* = 1.83) than before (*M* = 6.07, *SD* = 1.75), *F(*1, 140) = 31.79, *p* < *0.0*01, *f* = 0.47 (top panel of Fig. 5).

There was no significant interaction effect, *F(*1, 140) = 0.304, *p* = 0.582, η_p_^2^ < 0.01, and no significant main effect of condition, *F(*1, 140) = 2.62, *p* = 0.11, η_p_^2^ = 0.02. These results replicate—with a larger sample—a finding of Study 1 that the educational materials on alternative causes failed to significantly reduce the symptom discounting effect among problem-drinkers.

Participant ratings of their likelihood of becoming an alcoholic were also examined (bottom panel of Fig. 5). A mixed-design ANOVA revealed that problem-drinkers felt significantly less likely to become an alcoholic after being told that they lacked a genetic predisposition (*M* = 59.93, *SD* = 29.21) than before (*M* = 69.44, *SD* = 28.36), *F*(1, 140) = 43.95, *p* < 0.001, *f* = 0.55. There was no main effect of condition, *p* = 0.690, η_p_^2^ < 0.01, nor an interaction effect, *p* = 0.776, η_p_^2^ < 0.01.

### Other analyses

See Supplementary Information, Sect. 2 for analyses comparing non-problem-drinkers and problem-drinkers, which explore whether there are differences between these two groups in recognizing the symptom ramifications as well as in the efficacy of the educational materials. Any departure from the pre-registered analysis plan is explained in Supplementary Information, Sect. 3.

## Study 3

So far, Studies 1 and 2 have demonstrated that people experience the symptom discounting effect in relation to AUD. Furthermore, these studies failed to find that the educational materials that are currently used by a popular DTC company can effectively prevent the symptom discounting effect among problem-drinkers. Taken together, these results point to a strong need to develop more effective intervention materials, such that negative results from AUD genetic tests do not become risk factors for AUD.

Earlier, we explained that the symptom discounting effect is a violation of the Causal Markov condition. This theoretical analysis suggests that the most straightforward way to prevent the violation is to teach people about the condition. Thus, Study 3 examines a second version of educational materials which explain the Causal-Markov condition in ways that are comprehensible to the public. In addition, Study 3 included both the No-Education and the Alternative-Cause conditions to compare the effect of the Causal-Markov educational materials against each. Study 3 was preregistered via the Open Science Framework repository (see osf.io/vecwh).

### Methods

Of the 1920 participants who completed the survey, 579 failed the manipulation check, as they selected that they were asked to imagine that they had (as opposed to did not have) genetic risks for AUD. These 579 individuals were eliminated from the data analyses. Of the remaining 1341 participants, 711 were non-problem-drinkers (54.3% female, mean age of 41.3 [*SD* = 13.4], 77.4% white, 61.4% with a bachelor’s or higher degree) and 630 were problem-drinkers (38.1% female; mean age of 37.4 [*SD* = 10.8], 79.8% white, 76.0% with a bachelor’s or higher degree.

Participants were randomly assigned to either the No-Education (*n* = 238 non-problem-drinkers and *n* = 219 problem-drinkers), the Alternative-Cause condition (*n* = 235 non-problem-drinkers and *n* = 205 problem-drinkers), or the Causal-Markov condition (*n* = 238 non-problem-drinkers and *n* = 206 problem-drinkers). The No-Education (*M* = 9.67, *SD* = 8.54), Alternative-Cause (*M* = 9.50, *SD* = 8.48), and Causal-Markov (*M* = 9.27, *SD* = 8.51) conditions did not differ significantly in AUDIT scores, *F*(2, 1338) = 0.26, *p* = 0.774, η_p_^2^ < 0.01.

Study design and procedures were the same as in Study 2 except that there was an additional condition called the Causal-Markov condition. The educational materials used in the Causal-Markov condition were created by adding explanations about the Causal Markov principle to the educational materials used in the Alternative-Cause condition, because explaining the Causal Markov condition requires that one understand that alternative causes are present. That is, after learning that there are both genetic and non-genetic factors, participants learned that the ramifications of certain outcomes remain the same, regardless of what caused the outcome. For instance, they were told, “Even though your test results did not reveal a genetic predisposition for alcoholism, the problems that arise from alcoholism can happen regardless of whether one’s alcoholism was caused by genetic risk factors, or by other factors including one’s environment or lifestyle.” The Causal Markov condition was also explained using the example of how grass can be slippery when it is wet regardless of whether it became wet due to rain or a sprinkler. Then, they were told, “Likewise, a genetic predisposition to alcoholism is one of many causes for developing symptoms of alcoholism. And once someone develops symptoms of alcoholism, they can cause various negative consequences, including health problems and dysfunction in work or interpersonal relationships, regardless of how the person developed those symptoms in the first place.” These additional intervention instructions and the figures used to illustrate the Causal Markov condition are shown in Supplementary Information, Sect. 4.

## Results

The data were analyzed following the pre-registered plan. Cronbach’s alpha was high for both the 10 pre-test items (0.87) and the 10 post-test items (0.90). Thus, AUD symptom ramification scores for each participant were computed as in Study 1.

### Non-problem-drinkers

**Figure 6:**
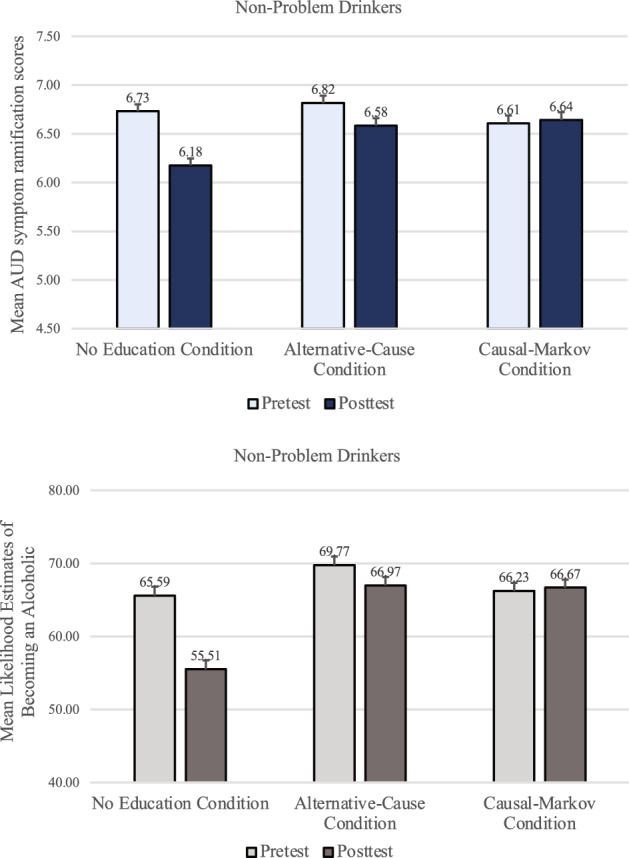
Non-problem-drinkers’ mean AUD symptom ramification scores and mean likelihood estimates of becoming an alcoholic in Study 3. Error bars represent *SEM* ^*pairedDiff*^.

A mixed-design timepoint X condition ANOVA examining AUD symptom ramification scores among non-problem-drinkers (top panel of Fig. 6) revealed that participants judged the AUD symptoms to be less dangerous after they imagined the lack of genetic predisposition (*M* = 6.47, *SD* = 1.92) than before (*M* = 6.72, *SD* = 1.78), *F*(1, 708) = 32.83, *p* < .001, *f* = .21. There was also a significant interaction effect, *F*(2, 708) = 15.17, *p* < .001, *η*_*p*_^2^ = .04. The main effect of condition was not significant, *p* = .292, *η*_*p*_^2^ < .01.

Paired *t*-tests indicated that non-problem-drinkers who did not receive any educational materials showed the discounting effect (*M* = 6.73, *SD* = 1.83 and *M* = 6.18, *SD* = 1.91 for before and after the genetic feedback, respectively), *t*(237) =  − 7.87, *p* < *0.0*01, *d* = 0.50. Unlike in Studies 1 and 2, however, the educational materials about alternative causes failed to prevent the discounting effect even among non-problem-drinkers. Their AUD symptom ramification scores in the Alternative-Cause condition were significantly lower after the genetic feedback (*M* = 6.58, *SD* = 1.82) than before (*M* = 6.82*, SD* = 1.65), *t*(234) =  − 3.07, *p* = 0.002, *d* = 0.21, underscoring the insufficiency of the alternative cause materials. Yet, the educational materials based on the Causal Markov assumptions successfully prevented non-problem-drinkers from discounting the AUD symptoms after the feedback (*M* = 6.61, *SD* = 1.84 for pre-test and *M* = 6.64, *SD* = 1.99 for post-test), *t*(237) = 0.413, *p* = 0.680, *d* = 0.03.

Participants’ ratings of their likelihood of becoming an alcoholic also showed a similar pattern. A mixed-design timepoint X condition ANOVA revealed that non-problem-drinkers judged that they were significantly less likely to become an alcoholic after being told that they lacked a genetic predisposition (*M* = 63.04, *SD* = 29.82) than before (*M* = 67.19, *SD* = 30.51), *F*(1, 706) = 38.22, *p* < *0.0*01, *f* = 0.23. There was a significant main effect of condition, *F*(2, 706) = 4.81, *p* = 0.008, η_p_^2^ = 0.01, and a significant interaction effect, *F*(2, 706) = 21.57, *p* < *0.0*01, η_p_^2^ = 0.06. As with the AUD symptom ramification scores, non-problem-drinkers in both the No-Education and the Alternative-Cause conditions felt significantly less likely to become an alcoholic after the feedback (*M* = 55.51, *SD* = 30.61, and *M* = 66.97, *SD* = 28.31, respectively) than before (*M* = 65.59, *SD* = 31.50, and *M* = 69.77, *SD* = 29.52, respectively), *t*(236) = 8.27, *p* < *0.0*01, *d* = 0.54 and *t*(234) = 2.42, *p* = 0.016, *d* = 0.16, respectively. Yet, their likelihood ratings in the Causal-Markov condition did not significantly decrease after the feedback (*M* = 66.67, *SD* = 29.15) than before (*M* = 66.23, *SD* = 30.44), *t*(236) =  − 0.40, *p* = 0.692, *d* = 0.03.

### Problem-drinkers

**Figure 7:**
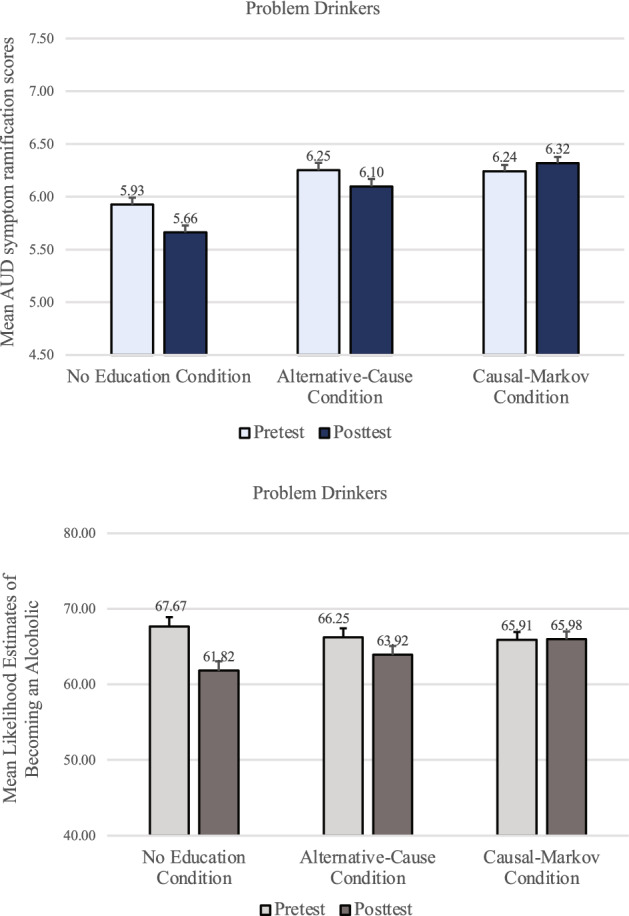
Problem-drinker’s mean AUD symptom ramification scores and mean likelihood estimates of becoming an alcoholic in Study 3, by condition at pre-test and post-test. Error bars represent *SEM* ^*pairedDiff*^.

For problem-drinkers, a mixed-design timepoint X condition ANOVA revealed that AUD symptom ramification scores (top panel of Fig. 7) were significantly lower after the genetic feedback (*M* = 6.02, *SD* = 1.67) than before (*M* = 6.14, *SD* = 1.51), *F(*1, 627) = 9.30, *p* = 0.002, *f* = 0.11. There was also a significant main effect of condition, *F*(2, 627) = 6.19, *p* = 0.002, η_p_^2^ = 0.02, and a significant interaction effect, *F(*2, 627) = 7.24, *p* < 0.001, η_p_^2^ = 0.02.

In the No-Education condition, problem-drinkers showed the discounting effect (*M* = 5.93, *SD* = 1.56 and *M* = 5.66, *SD* = 1.76 for before and after the genetic feedback, respectively), *t*(218) =  − 4.03, *p* < *0.0*01, *d* = 0.27. In the Alternative-Cause condition, replicating Studies 1 and 2, problem-drinkers still discounted the AUD symptoms after the feedback (*M* = 6.10, *SD* = 1.62) compared to before (*M* = 6.25, *SD* = 1.47), *t*(204) =  − 2.18, *p* = 0.030, *d* = 0.15, despite the educational materials. In the Causal-Markov condition, however, there was no difference in AUD symptom ramification scores before (*M* = 6.24, *SD* = 1.49) and after the feedback (*M* = 6.32, *SD* = 1.55), *t*(205) = 1.33, *p* = 0.186, *d* = 0.09. Participants’ ratings of the likelihood of becoming an alcoholic were also examined. A mixed-design timepoint X condition ANOVA revealed that problem-drinkers felt significantly less likely to become an alcoholic after being told that they lacked a genetic predisposition (*M* = 63.87, *SD* = 25.57) than before (*M* = 66.63, *SD* = 25.48), *F*(1, 625) = 16.63, *p* < *0.0*01, *f* = 0.16. There was no significant main effect of condition, *p* = 0.872, but a significant interaction effect, *F*(2, 625) = 6.82, *p* = 0.001, η_p_^2^ = 0.02.

As with the AUD symptom ramification scores, problem-drinkers in both the No-Education and the Alternative-Cause condition felt significantly less likely to become an alcoholic after the feedback (*M* = 61.82, *SD* = 26.66 and *M* = 63.92, *SD* = 25.02, respectively) than before (*M* = 67.67, *SD* = 25.95 and *M* = 66.25, *SD* = 25.03, respectively), *t*(216) = 4.74, *p* < *0.0*01, *d* = 0.32 and *t*(204) = 2.01, *p* = 0.046, *d* = 0.14, respectively. Yet, in the Causal-Markov condition, the likelihood estimates before the feedback (*M* = 65.91, *SD* = 25.51) did not differ from those after the feedback (*M* = 65.98, *SD* = 24.88), *t*(205) =  − 0.071, *p* = 0.943, *d* < 0.01.

### Other analyses

See Supplementary Information, Sect. 2 for analyses comparing non-problem-drinkers and problem-drinkers, which explore whether there are differences between these two groups in recognizing the symptom ramifications as well as in the efficacy of the educational materials.

## Discussion

Previous studies have shown that those who learned that they had genetic risks of alcoholism reported feeling less control over their drinking than those who did not^9^. The current research found for the first time that learning that one lacks a genetic predisposition to AUD can also have negative effects by potentially leading people to take symptoms of AUD less seriously and to underappreciate the potential negative consequences of alcohol overuse.

In Study 1, participants rated their reactions to the same AUD symptoms before and after being told that they were not genetically predisposed to AUD. To avoid the selection bias of relying on data only from those who trusted this sham feedback, participants in Studies 2 and 3 were told to imagine that they lacked a genetic predisposition to AUD, although merely imagining this feedback could be a weaker experimental manipulation than seeing one’s own saliva changing the color of a test strip. Across all three experiments, despite the symptoms being identical and despite that the pre-test ratings could have prevented people from changing their post-test ratings in order to be consistent, the negative feedback caused both problem-drinkers and non-problem-drinkers to treat the symptoms less seriously, to assume that it was less urgent to seek treatment or to try to change their behaviors, and to believe that their drinking would be less harmful to their work and social functioning. These findings have immediate, real-life implications, as people who learn that they lack a genetic predisposition to AUD might be less likely to seek professional treatment for themselves or their family members if symptoms emerge. Furthermore, this symptom discounting effect may lead people to feel entitled to drink more.

The current research also tested the effects of presenting educational materials. One version of these materials was based on the FDA-approved approaches already used by a DTC genetic testing company, where participants learned that people who test negative for a genetic predisposition can still be at risk of AUD. While these educational materials counteracted the symptom discounting effect among non-problem-drinkers in Studies 1 and 2, they failed to do so for problem-drinkers in all three studies. Despite the thorough explanations and warnings about AUD in these materials, the problem-drinkers still discounted the dangers and urgency of AUD symptoms significantly more after imagining or being told that they were not genetically predisposed to AUD. The failure of these materials is particularly alarming as problem-drinkers are at higher risk for AUD, and discounting the seriousness of the AUD symptoms could be more harmful for these people. Furthermore, in Study 3, this intervention was not effective in dispelling the symptom discounting effect even among non-problem-drinkers.

We then tested a stronger intervention method, based on our analysis that the symptom discounting effect is a violation of the Causal Markov condition. Specifically, participants were taught that once AUD symptoms are present, their implications are the same, regardless of what caused those symptoms in the first place. This new approach successfully removed the symptom discounting effect among both the non-problem-drinkers and problem-drinkers.

The fact that this Causal-Markov intervention was effective suggests that a psychological mechanism underlying the symptom discounting effect is a tendency among laypeople, especially problem-drinkers, to misrepresent the causal structure such that they interpreted AUD symptoms caused by genes to be different and more concerning, compared to AUD symptoms caused by non-genetic factors. By helping them revise this misconception, the current study identified the first empirically validated method of dispelling the symptom discounting effect.

There are also some limitations of the current findings. As explained earlier, we used sham genetic test results to draw causal conclusions about the role of genetic feedback in inducing the symptom discounting effect. Experimentally manipulating genetic feedback eliminates potential selection bias (e.g., those who spontaneously seek real-life health-related genetic testing may be particularly health-conscious). Despite these advantages, the current findings should be replicated using actual genetic test results, as FDA-approved DTC screening for genetic risks for AUD becomes available in the near future. Further research could also examine the durability of the symptom discounting effect and of the Causal-Markov educational materials.

Another potential limitation of the current research is that our dependent measures were self-report items rather than changes in subsequent drinking behaviors. The use of the sham saliva test feedback did not allow us to observe actual drinking patterns, because it would be unethical to prolong the period of deception. On the one hand, self-reported attitudes are a strong predictor of alcohol-use intentions, which in turn are a strong predictor of alcohol-use behaviors as shown by meta-analysis^42^. On the other hand, future studies could examine how learning that one lacks a genetic predisposition to AUD affects subsequent alcohol use and treatment-seeking, while following up with those who test negative during actual DTC genetic testing.

Testing negative for health-related genetic risk is welcome news for anybody. Nonetheless, the current study found perils of receiving such good news that the public should be aware of so that the test results themselves do not become risk factors. The educational materials demonstrated to be effective in the present study should be considered for adoption in DTC genetic testing for health conditions.

## Supplementary Information

Supplementary Information.

## Data Availability

All data used for the analyses in Studies 1–3 are deposited at https://osf.io/t524u/files.
