# Supplementary material for: The symptom discounting effect: what to do when negative genetic test results become risk factors for alcohol use disorder
Source: Sci Rep. 2022 Mar 4;12:3579. doi: 10.1038/s41598-022-07452-5 (PMC8897420; doi:10.1038/s41598-022-07452-5)
Supplement: Supplementary file 1 — Supplementary Information. [file 41598_2022_7452_MOESM1_ESM.docx]

**Supplementary Information**

**Section1:** **Human Subjects Protection**

**Procedures taken to minimize risk to participants**

Because the procedures of Study 1 involved deception, this section details the methods used to protect human subjects from being harmed as a result. All procedures were developed through careful assessment of risks and burdens to the participants and were approved by the Institutional Review Board.

Firstly, the informed consent document explained that participants could withdraw at any time without losing compensation. Participants were also informed that if they did wish to withdraw prematurely, they should do so by using the “exit study” link provided on every page of the Qualtrics.com program, which ensured that they would immediately view the debriefing form (see below). Specifically, they were told, “If you would like to discontinue your participation in this study, you may click on the blue "Exit Study" link in the bottom left corner of each page. If you do wish to exit the study early, it is important that you click the "Exit Study" link instead of merely closing the browser window (exiting the study via this link will also allow you to still be compensated as a study participant). Be careful! Once you click this button you cannot return to the survey.” As reported in the main text, no participant ended the study without having received the debriefing after having accessed the deceptive portion of the study.

In total, the deception lasted less than ten minutes before participants were made aware via the debriefing form that the saliva test used in the study was a sham. Part of the debriefing form (i.e., explanations of the perseverance effect in the debriefing in the next section) was developed based on the literature on effective debriefing methods for experiments involving deception^[[1]](#footnote-1)^. To ensure that participants read and comprehended the debriefing form, three true/false questions were added at the end (see below), which were repeated if participants answered any of the questions incorrectly. All participants eventually answered all of these questions correctly. There were no reports of adverse effects to the study investigators or the Yale University IRB. Previous studies used the same saliva test procedures and similar debriefing forms and also had no reports of adverse events^[[2]](#footnote-2)^.

**Verbatim debriefing form**

| Thank you for your participation in this study. This document contains important information that is relevant to the current study.  Please read the information below carefully.  At the bottom of this page, there are True/False questions about the information below. You must answer these questions correctly in order to complete this study.  The study in which you have participated is about people’s reactions to receiving personalized genetic information related to their risk of a particular health condition. Although participants in the study were told that information about their genetic makeup was revealed by their answers to certain questions or through a biochemical test of their saliva, this is NOT TRUE. NO ACTUAL GENETIC TESTING WAS CONDUCTED AS PART OF THIS STUDY AND NO INFORMATION ABOUT PARTICIPANTS’ GENETIC MAKEUP WAS TRULY REVEALED BY ANSWERING THE QUESTIONS ASKED IN THIS STUDY.  It is very important for you to know that:   - YOUR CELLS/TISSUES HAVE NOT BEEN TESTED TO DETERMINE YOU GENETIC MAKEUP as part of this study. Additionally, THE QUESTIONS ASKED IN THIS STUDY HAVE NOT ACTUALLY BEEN SHOWN TO REVEAL ANYTHING SPECIFIC ABOUT PEOPLE’S GENETIC RISK FOR ANY HEALTH CONDITION. - As a result, THIS STUDY HAS NOT REVEALED ANY ACTUAL INFORMATION ABOUT YOUR TRUE GENETIC MAKEUP OR YOUR RISK FOR ANY MEDICAL OR PSYCHIATRIC PROBLEM.    The saliva test you received is actually a glucose test strip designed for use by individuals with diabetes, and since the mouthwash used with the strip in our study contains glucose, this causes the test strip to change color in reaction to participants' saliva. We produced the labels for the plastic container so that the kit would look like a genuine test.  Temporarily altering people’s beliefs about their genetic susceptibility to health problems is part of this study because we are attempting to learn how people react to receiving personalized genetic information. Our hypothesis is that being led to believe they have reduced genetic risk of a certain health problem may lead people to feel somewhat “invincible,” which could lead to problematic health beliefs and behaviors. This is troubling because genetic tests that claim to inform people of their risk for health problems are likely to become a common part of healthcare in the near future. Thus, it is important to understand how receiving such information affects people psychologically.  In addition, when people participate in studies like this one in which they are given bogus information, sometimes the beliefs created by that bogus information persist even after debriefing because the participants have already generated independent evidence that explains the feedback. For instance, if you were told that you lacked a genetic predisposition toward alcoholism in our fake genetic test, you may have recalled a time when you drank more heavily without becoming an alcoholic, and you may have attributed this outcome to your genes. Once you attempt to generate such explanations, those incidents may stick in your mind as evidence, and thus even after you are told that the genetic testing was fake, your belief in the bogus test result you were given may persevere. This so-called perseverance effect is irrational, and you should avoid thinking in this way.  You have the option to withdraw from the experiment at this point if you wish. If you would prefer that the information you provided today not be included in the study data, please notify the experimenter.  If you are experiencing problems with alcoholism, or experiencing any other psychological difficulty, you should know that treatment is available and may benefit people who are experiencing psychological difficulties.  Mental health treatment providers in your area can be found at: http://www.abct.org/Members/?m = FindTherapist&fa = FT_Form  Additionally, if you are currently in crisis, you can call 800-273-8255 to speak to a trained counselor.  IF YOU FEEL YOU ARE IN IMMEDIATE DANGER, PLEASE CALL 911.  Thank you again for participating in this study. If you would like further information or have any questions later, please e-mail woo.kyoung.ahn@yale.edu.   If you have any questions about your rights as a research participant or concerns about the conduct of this study, you may contact the Yale University Human Subjects Committee, Box 208010, New Haven, CT 06520-8010, 203-785-4688, human.subjects@yale.edu. Additional information is available at http://www.yale.edu/hrpp/participants/index.html  Please answer the true/false questions below:   - No information about your actual genetic makeup was revealed as part of the experiment in which you participated today. (True/False) - The procedures of this study did not actually reveal any information about your biological risk for any health problem. (True/False) - Treatment is available for people who are experiencing psychological distress. (True/False) |
| --- |

**Section 2: Comparing Problem vs. Non-problem drinkers**

The main text reports results from separate analyses for non-problem-drinkers and problem-drinkers. This section provides analyses comparing non-problem-drinkers and problem-drinkers to explore whether there are differences between these two groups in recognizing the symptom ramifications as well as in the efficacy of the educational materials. The results from 2 (drinking: problem vs. non-problem drinkers) X timepoint (pre-test vs. post-test) ANOVAs within each condition in each study on their symptom ramification scores are shown in the tables on the next page. Below is a summary of the results.

a. Problem-drinkers rated symptom ramifications to be lower than non-problem drinkers across all conditions of all studies, regardless of the timepoint, as shown by the significant main effects of drinking.

b. In the no-education conditions, the amount of discounting caused by the genetic feedback (i.e., the difference between the post-test and pre-test) did not depend on whether participants were problem or non-problem drinkers in Studies 1 and 2 as shown by the lack of significant interaction effects in these conditions. That is, both groups’ symptom ramification scores were lowered to a similar extent after the genetic feedback without the educational materials. The amount of symptom discounting was greater among the non-problem drinkers than problem-drinkers in the no-education condition of Study 3. It is not clear why that might have been the case, but either way, there is no evidence suggesting that the symptom discounting without education materials is greater among problem drinkers.

In interpretating these results, it should be noted that the AUDIT scores of our participants are heavily skewed towards the lower end, which is why regression analyses treating AUDIT scores as a continuous variable are not performed. It is possible that the symptom discounting effect could be more pronounced with larger samples of problem drinkers with extremely high AUDIT scores.

c. Consistent with the results reported in the main text, the alternative-cause materials counteracted discounting caused by the genetic feedback among the non-problem-drinkers more than among the problem-drinkers in Studies 1 and 2, as shown by the significant interaction effects in the Alternative-Cause condition of these two studies. In Study 3, however, both groups of participants were equally unaffected by the alternative-cause materials, as shown by the lack of significant interaction effect and the significant main effect of timepoint.

d. The Causal-Markov materials in Study 3 similarly affected problem and non-problem drinkers.

| **No-Education condition** | | Study 1 | | | Study 2 | | | Study 3 | | |
| --- | --- | --- | --- | --- | --- | --- | --- | --- | --- | --- |
|  |  | Pre-test | Post-test | Total | Pre-test | Post-test | Total | Pre-test | Post-test | Total |
| Non-Problem Drinkers | M | 6.66 | 6.39 | 6.52 | 6.73 | 6.20 | 6.46 | 6.73 | 6.18 | 6.45 |
|  | SE | 0.21 | 0.22 | 0.20 | 0.13 | 0.15 | 0.13 | 0.12 | 0.12 | 0.11 |
| Problem-Drinkers | M | 5.26 | 4.92 | 5.09 | 6.32 | 5.74 | 6.03 | 5.93 | 5.66 | 5.79 |
|  | SE | 0.22 | 0.22 | 0.21 | 0.20 | 0.23 | 0.20 | 0.11 | 0.12 | 0.12 |
| Total | M | 5.96 | 5.66 |  | 6.52 | 5.97 |  | 6.33 | 5.92 |  |
|  | SE | 0.15 | 0.16 |  | 0.12 | 0.13 |  | 0.08 | 0.09 |  |
| Main effect of Timepoint | | *F*(1,146) = 10.77, *p* = .001, *f* = .26 | | | *F*(1,227) = 42.09, *p* < .001, *f* = .42 | | | *F*(1,455) = 71.693, *p* < .001, *f* = .39 | | |
| Main effect of Drinking | | *F*(1,146) = 24.28, *p* < .001, n_p_^2^ = .14 | | | *F*(1,227) = 3.34, *p* = .069, n_p_^2^ = .01 | | | *F*(1,455) = 17.22, *p* < .001, n_p_^2^ = .04 | | |
| Interaction effect | | *F*(1,146) = .17, *p* = .685, n_p_^2^ < .01 | | | *F*(1,227) = .08, *p* = .774, n_p_^2^ < .01 | | | *F*(1,455) = 9.00, *p* = .003, n_p_^2^ = .02 | | |

| **Alternative-Cause condition** | | Study 1 | | | Study 2 | | | Study 3 | | |
| --- | --- | --- | --- | --- | --- | --- | --- | --- | --- | --- |
|  |  | Pre-test | Post-test | Total | Pre-test | Post-test | Total | Pre-test | Post-test | Total |
| Non-Problem Drinkers | M | 6.57 | 6.78 | 6.68 | 7.09 | 6.96 | 7.02 | 6.82 | 6.58 | 6.70 |
|  | SE | 0.20 | 0.21 | 0.21 | 0.13 | 0.13 | 0.13 | 0.11 | 0.12 | 0.10 |
| Problem Drinkers | M | 5.44 | 5.24 | 5.34 | 5.81 | 5.33 | 5.57 | 6.25 | 6.10 | 6.18 |
|  | SE | 0.23 | 0.25 | 0.22 | 0.21 | 0.20 | 0.20 | 0.10 | 0.11 | 0.11 |
| Total | M | 6.01 | 6.01 |  | 6.45 | 6.14 |  | 6.53 | 6.34 |  |
|  | SE | 0.15 | 0.16 |  | 0.12 | 0.12 |  | 0.08 | 0.08 |  |
| Main effect of Timepoint | | *F*(1,151) = .001, *p* = .979, *f* < .001 | | | *F*(1,230) = 16.81, *p* < .001, *f* = .26 | | | *F*(1,438) = 13.70, *p* < .001, *f* = .17 | | |
| Main effect of Drinking | | *F*(1,151) = 19.46, *p* < .001, n_p_^2^ = .11 | | | *F*(1,230) = 38.84, *p* < .001, n_p_^2^ = .14 | | | *F*(1,438) = 12.42, *p* < .001, n_p_^2^ = .03 | | |
| Interaction effect | | *F*(1,151) = 5.74, *p* = .018, n_p_^2^ = .04 | | | *F*(1,230) = 5.48, *p* = .020, n_p_^2^ = .02 | | | *F*(1,438) = .56, *p* = .455, n_p_^2^ < .01 | | |

| **Causal-Markov condition,** Study 3 | | Pre-test | Post-test | Total |  |  |
| --- | --- | --- | --- | --- | --- | --- |
| Non-Problem Drinkers | M | 6.61 | 6.64 | 6.62 | Main effect of Timepoint | *F*(1,442) = 1.15, *p* = .285, *f* = .02 |
|  | SE | 0.12 | 0.13 | 0.11 | Main effect of Drinking | *F*(1,442) = 4.73, *p* = .030, n_p_^2^ = .01 |
| Problem Drinkers | M | 6.24 | 6.32 | 6.28 | Interaction effect | *F*(1,442) = .17, *p* = .678, n_p_^2^ < .01 |
|  | SE | 0.10 | 0.11 | 0.12 |  |  |
| Total | M | 6.43 | 6.48 |  |  |  |
|  | SE | 0.08 | 0.09 |  |  |  |

**Section 3: Deviations from the Pre-registered Analysis Plan for Study 2**

The pre-registered analysis plan for Study 2 was to conduct mixed-design ANCOVA’s on post-test scores, treating conditions as a between-subjects factor and timepoint as a within-subjects variable controlling for pre-test scores across all participants, and then separately for the problem drinkers and the non-problem drinkers. In addition, it was planned to conduct independent samples *t*-tests comparing the magnitude of difference scores (post-test minus pre-test scores) in the two conditions – which is mathematically identical to conducting the mixed-design ANOVA reported in the main text. As with the ANCOVAs, the plan was to carry out these *t*-tests over difference scores across all participants and also separately for the problem drinkers and the non-problem drinkers.

Upon discovering that the pattern of results differs between the problem drinkers and the non-problem drinkers in Study 2 as well as in Study 1, we have decided to report only the results broken down by problem-drinkers and non-problem drinkers, as any analyses overlooking this empirically as well as clinically important distinction would be rather misleading. In addition, ANCOVA’s and the mixed-design ANOVA’s reported in the main text yielded similar results, so to avoid redundance, only the results from mixed-design ANOVA’s are reported.

Finally, the analyses reported in Sections 2 and 5 of Supplementary Information were not pre-registered, and were added during the review process.

**Section 4: Causal-Markov Educational Materials**

Each table cell corresponds to a separate page of text in the Qualtrics program used to administer all study procedures.

**Part 1**

(Part 1 educational materials used in the Alternative-cause condition were presented first.)

Furthermore, alcoholism affects people in various ways. People with alcoholism may experience increased health risks (such as liver disease and various types of cancers), problems at work, financial problems, unemployment, as well as problems in their interpersonal relationships (such as family disruption). Once drinking becomes uncontrollable, it can also lead to even more excessive drinking.

**Part 2**

(Part 2 educational materials used in the Alternative-cause condition were presented first.)

| Earlier, we also explained that alcoholism can interfere with work or social functioning and cause other health risks.  Even though your test results did not reveal a genetic predisposition for alcoholism, the problems that arise from alcoholism can happen regardless of whether one’s alcoholism was caused by genetic risk factors, or by other factors including one’s environment or lifestyle. That is, once one starts showing signs of alcoholism, the consequences can be serious and harmful no matter whether the person developed alcoholism because of their genetics or because of non-genetic factors. |
| --- |
| One way to think about this is as follows. We know that grass can get wet when it rains or when a sprinkler is turned on. We also know that when grass is wet, grass becomes slippery. This relationship is illustrated in the figure below.   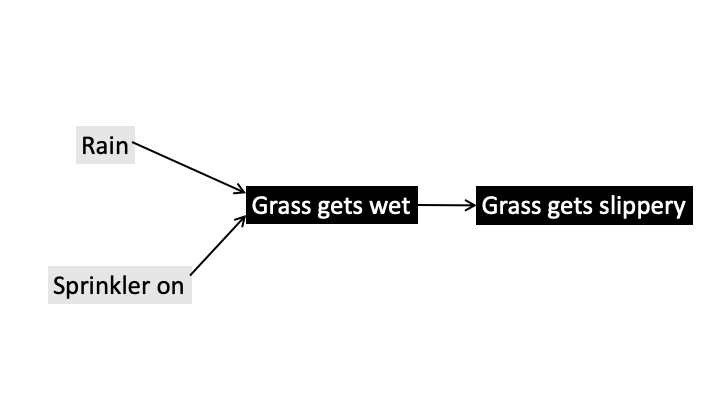 Once we find out that grass is wet, we can assume that the grass would be slippery. And the extent to which the grass is slippery would be the same, no matter whether the grass became wet because of rain or because of a sprinkler. Thus, regardless of what caused the grass to get wet, we would be careful about walking on the grass. |
| Likewise, a genetic predisposition to alcoholism is one of many causes for developing symptoms of alcoholism. And once someone develops symptoms of alcoholism, they can cause various negative consequences, including health problems and dysfunction in work or interpersonal relationships, regardless of how the person developed those symptoms in the first place. Thus, once a person shows signs of alcoholism, they should take these symptoms seriously no matter whether they developed the symptoms because of their genes or for other reasons. 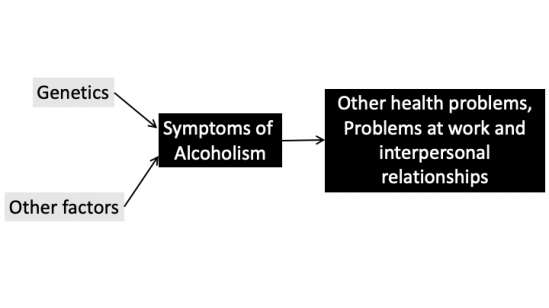 |

**Section 5. The Symptom Discounting Effect for Each of the 10 Items Used in Studies 1-3**

In assessing the symptom discounting effect, the analyses reported in the main text used ratings collapsed across 10 items (see Table 1 of the main text) as they showed high inter-item reliability. We additionally tested the differences between the pre-test and post-test ratings for each of these 10 items across all the no-education conditions of Studies 1-3. The following table summarizes the mean differences, SE’s, and statistics from the paired *t*-tests. The item number corresponds to the numbers in Table 1.

| Item Number | Abbreviated Descriptions | Mean Differences (Pretest - Posttest ratings) | SE | t | df | p |
| --- | --- | --- | --- | --- | --- | --- |
| 1 | Self-Seriousness | 0.612 | 0.075 | 8.104 | 833 | <.001 |
| 2 | Self-Confidence | 0.284 | 0.071 | 3.979 | 833 | <.001 |
| 3 | Self-Urgency | 0.265 | 0.073 | 3.647 | 833 | <.001 |
| 4 | Family Seriousness | 0.559 | 0.061 | 9.117 | 833 | <.001 |
| 5 | Family Confidence | 0.561 | 0.076 | 7.422 | 833 | <.001 |
| 6 | Family Urgency | 0.446 | 0.064 | 7.003 | 833 | <.001 |
| 7 | Need to Change Drinking | 0.489 | 0.073 | 6.687 | 833 | <.001 |
| 8 | Need to Stop Drinking | 0.429 | 0.071 | 6.079 | 833 | <.001 |
| 9 | Harms | 0.296 | 0.07 | 4.217 | 833 | <.001 |
| 10 | Functioning | 0.369 | 0.066 | 5.605 | 833 | <.001 |

1. L. Ross, M. R. Lepper, M. Hubbard, Perseverance in self-perception and social perception: biased attributional processes in the debriefing paradigm. *Journal of Personality and Social Psychology* **32**, 880 (1975).

   C. McFarland, A. Cheam, R. Buehler, The perseverance effect in the debriefing paradigm: Replication and extension. *Journal of Experimental Social Psychology* **43**, 233-240 (2007). [↑](#footnote-ref-1)
2. Lebowitz, M. S. & Ahn, W. Blue genes? Understanding and mitigating negative consequences of personalized information about genetic risk for depression. *Journal of Genetic Counseling* **27**, 204-216 (2018).

   Ahn, W. & Lebowitz, M. S. An experiment assessing effects of personalized feedback about genetic susceptibility to obesity on attitudes towards diet and exercise. *Appetite* **120**, 23-31, doi:<https://doi.org/10.1016/j.appet.2017.08.021> (2018).

   Lebowitz, M. S., & Ahn, W. K. (2017). Testing positive for a genetic predisposition to depression magnifies retrospective memory for depressive symptoms. *Journal of consulting and clinical psychology*, *85*(11), 1052. [↑](#footnote-ref-2)
